# Altered secretion patterns and cell wall organization caused by loss of PodB function in the filamentous fungus *Aspergillus nidulans*

**DOI:** 10.1038/s41598-018-29615-z

**Published:** 2018-07-30

**Authors:** Karthik R. Boppidi, Liliane Fraga Costa Ribeiro, Sirasa Iambamrung, Sidney M. Nelson, Yan Wang, Michelle Momany, Elizabeth A. Richardson, Stephen Lincoln, Ranjan Srivastava, Steven D. Harris, Mark R. Marten

**Affiliations:** 1 University of Maryland - Baltimore County, Department of Chemical Biochemical and Environmental Engineering, Baltimore, MD USA; 2 University of Maryland – College Park, Department of Cell Biology and Molecular Genetics, College Park, MD USA; 3 University of Georgia, Fungal Biology Group and Department of Plant Biology, Athens, GA USA; 4 University of Georgia, Georgia Electron Microscopy, Athens, GA USA; 5 University of Connecticut, Department of Chemical and Biomolecular Engineering, Storrs, CT USA; 6 University of Manitoba, Department of Biological Sciences, Winnipeg, MB Canada

## Abstract

Filamentous fungi are widely used in the production of a variety of industrially relevant enzymes and proteins as they have the unique ability to secrete tremendous amounts of proteins. However, the secretory pathways in filamentous fungi are not completely understood. Here, we investigated the role of a mutation in the POlarity Defective (*podB*) gene on growth, protein secretion, and cell wall organization in *Aspergillus nidulans* using a temperature sensitive (Ts) mutant. At restrictive temperature, the mutation resulted in lack of biomass accumulation, but led to a significant increase in specific protein productivity. Proteomic analysis of the secretome showed that the relative abundance of 584 (out of 747 identified) proteins was altered due to the mutation. Of these, 517 were secreted at higher levels. Other phenotypic differences observed in the mutant include up-regulation of unfolded protein response (UPR), deformation of Golgi apparatus and uneven cell wall thickness. Furthermore, proteomic analysis of cell wall components in the mutant revealed the presence of intracellular proteins in higher abundance accompanied by lower levels of most cell wall proteins. Taken together, results from this study suggest the importance of PodB as a target when engineering fungal strains for enhanced secretion of valuable biomolecules.

## Introduction

Filamentous fungi are widely used as cellular factories in the bioprocess industry for the production of many important products including commodity chemicals, antibiotics, and enzymes^1^. Of particular significance is the ability of fungi to express and secrete both native and recombinant proteins^2^. However, while capable of secreting extraordinary amounts of native proteins, fungi are typically unable to produce and secrete appreciable amounts of non-fungal protein^3^. Strategies employed to improve non-fungal protein expression include both biological, i.e., genetic manipulation of host cells^3–6^, and engineering, i.e., modification of bioprocesses^7,8^ approaches. While these strategies have led to improvements, titers of non-fungal proteins are often 100-fold lower than those of native fungal proteins^1,9^. The reason for this disparity is not clear due to an incomplete understanding of protein secretory mechanisms and associated biological processes in filamentous fungi.

In filamentous fungi, proteins destined for secretion enter the secretory pathway at the endoplasmic reticulum (ER)^10^. The ER is able to balance translation of new proteins with processing capacity. Disruption of this balance causes an accumulation of mis-folded proteins, resulting in ER stress, and subsequently, activation of the unfolded protein response (UPR)^11^. Additionally, the UPR is reported to be induced when fungi experience conditions that require elevated levels of protein secretion^12^. Accordingly, improvement in protein secretion has been achieved by modifying components involved in ER stress response such as *A. niger hacA*^13^. Recently, increased protein secretion has also been achieved by deletion of *A. oryzae Vip36* and *Emp47* that lead to ER stress^14^. These findings suggest additional improvements may be possible through modification of other protein trafficking machinery in the filamentous fungal secretory pathway.

Recently, the A. *nidulans podB* gene has been identified as a homologue of the yeast (*S. cerevisiae*) *COG2* gene that encodes for a subunit of the conserved oligomeric complex of Golgi (COG)^15^. The *COG2* gene product is reported to be involved in the protein secretory pathway in S. *cerevisiae* - facilitating ER to Golgi vesicle docking^16–18^. In *A. nidulans*, PodB is reported to affect polar growth, germination, and glycosylation of proteins^15,19^. Protein glycosylation influences many biological processes in filamentous fungi including protein folding, oligomerization, sorting and transport^20^. Recently, it has been shown that deletion of *alg3*, a gene involved in N-glycosylation of proteins, leads to an increase in overall protein secretion^21^. As PodB is involved both in the trafficking of proteins and also protein glycosylation, we sought to better understand its role in the protein secretory pathway.

In this study, we find that an *A. nidulans* strain with a mutation (described as *podB1* mutation) in *podB* gene has an increased ability to secrete proteins when compared to a wild type control. Further, we studied the different types of proteins secreted by the mutant using quantitative proteomic analysis tools. In addition, as cell wall organization is a direct consequence of protein secretion^22,23^, we studied molecular changes in the abnormal cell wall of the mutant using cell wall proteomics. Finally, we report on the physiological implications and factors related to high protein secretion observed in mutant strain. These data collectively suggest that alteration of PodB function might represent a key step in the engineering of fungal strains for increased production of native and non-fungal proteins.

## Results

### Phenotypic effect of *podB1* mutation

In order to understand the effect of the *podB1* mutation, both ASH83 (which contains the *podB1* mutation) and A28 (control strain) were grown in MAG media in shake flasks. Temperature shift experiments were conducted, as ASH83 is a temperature sensitive (TS) mutant^15,19^. When grown at the permissive temperature (PT), conidia of both A28 and ASH83 germinated and then exhibited polar growth (Supplementary Fig. S1A,C respectively). However, when grown at the restrictive temperature (RT), ASH83 showed isotropic growth and undeveloped germ tubes (Supplementary S1D). This is in contrast to A28 which showed polar growth and fully developed hyphae when it grown at the restrictive temperature (Supplementary Fig. S1B).

### Genotype of the *podB1* mutation

It was previously reported that the phenotype observed in ASH83 was due to a G to T nucleotide substitution at position 670 of the *podB* open reading frame^15^. This mutation was predicted to create a stop codon that results in the truncation of the last 67 amino acids of the protein. However, upon re-sequencing the AN8226 (*podB*) gene from both the mutant and wild type strains, we found that position 670 was unchanged. Instead, the only mutation present in the mutant is a G to T nucleotide substitution at position 751 that also generates a stop codon (Supplementary Fig. S2). Accordingly, the last 67 amino acids of PodB would be truncated in the mutant.

### Effect of the *podB1* mutation on growth and protein productivity

**Figure 1:**
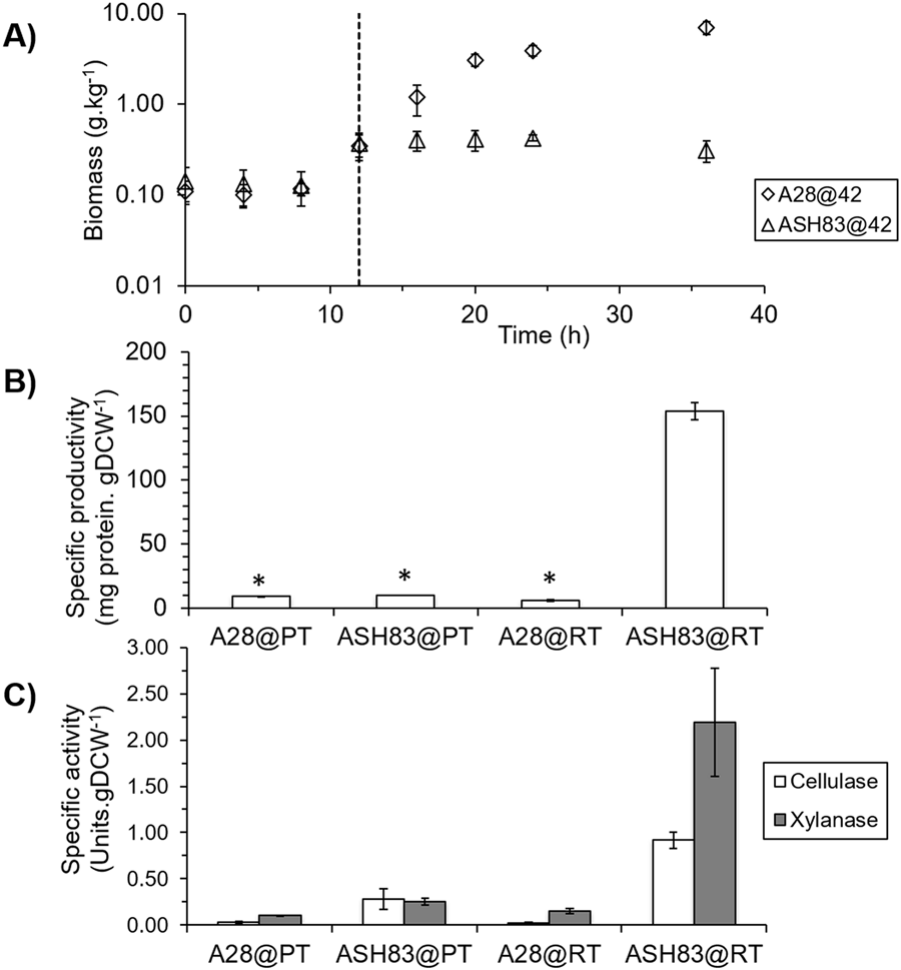
Effect of Ts *podB1* mutation on growth and protein productivity. (**A**) The biomass time course for A28 and ASH83 strains grown in YGV media at 28 °C for 12 h before shifting the temperature to 42 °C. The shift in temperature is shown by the vertical dotted line at 12 h. Temperature shift does not impact the growth of A28 but the biomass of ASH83 does not increase after the temperature is shifted to 42 °C. (**B**) Specific protein productivity of strains at both 28 °C and 42 °C at 36 h in liquid culture. Abbreviations: A28 grown only at 28 °C (A28@PT), ASH83 grown only at 28 °C (ASH83@PT), A28 grown at 28 °C before shifting to growth at 42 °C (A28@RT) and ASH83 grown at 28 °C before shifting to growth at 42 °C (ASH83@RT). There are no statistically significant differences between A28@PT, ASH83@PT and A28@RT. However, ASH83@RT shows 15X higher protein productivity compared to the other conditions. (n = 3, *p > 0.05). (**C**) Specific enzyme activity assays (cellulase, white bars; xylanase, grey bars) under same conditions in. (**B**) There were statistically no significant differences in the specific activities of A28@PT, ASH83@PT and A28@RT. But ASH83@RT had significantly higher specific cellulase and xylanase activity compared to the other conditions (n = 3).

A temperature shift strategy was utilized for both A28 and ASH83 strains to obtain adequate biomass for subsequent testing. Both strains were initially grown at the permissive temperature, i.e at 28 °C, for 12 hours and then shifted to restrictive temperature (42 °C) allowing the *podB1* phenotype to manifest. We observed no statistically significant differences in biomass between ASH83 and A28 during growth at 28 °C, but there are significant differences after the temperature shift to 42 °C (Fig. 1A). After the temperature shift A28 continued to grow as expected while biomass concentration of ASH83 remained unchanged. To determine the effect of the *podB1* mutation on protein secretion, we measured total protein secreted by both ASH83 and A28 strains after 36 h growth in liquid culture. We observed no statistically significant differences in total protein secreted between A28 and ASH83 at the permissive temperature (n = 3, p = 0.51). Interestingly, there was also no significant difference in total protein secreted between A28 and ASH83 at the restrictive temperature (n = 3, p = 0.14). While the ASH83 strain produced and secreted a similar, total amount of protein, we note there was a tremendous difference in specific protein productivity (mg protein secreted per gram biomass) for this strain. This difference in productivity occurred because ASH83 biomass did not increase after the temperature shift to 42 °C, but the strain continued to produce and secrete protein. As a result, specific productivity was 15 times higher in the ASH83 strain than in the control strain (Fig. 1B). And this difference was only evident at the restrictive temperature (42 °C), implying the difference was due to the *podB1* mutation. To determine if these secreted proteins retained their activity, we measured activities of cellulase and xylanase secreted by both strains. For both enzymes, we observed no significant differences until ASH83 was grown at 42 °C (Fig. 1C) where cellulase and xylanase activities were respectively 43 and 14.5 times greater than when A28 was grown at 42 °C.

### Effect of *podB1* mutation on the types of proteins secreted

**Figure 2:**
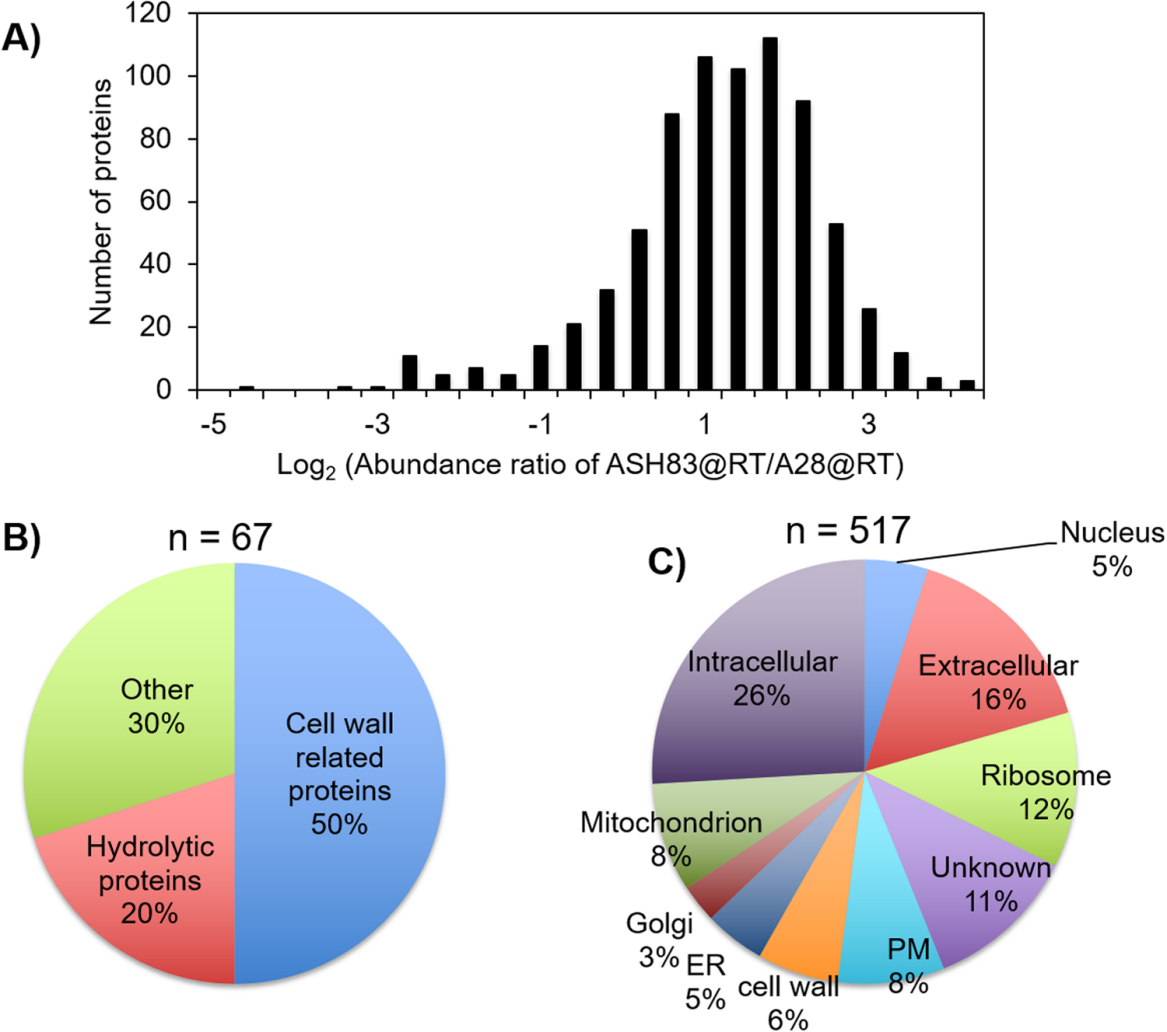
Quantitative proteomic analysis of secretome identifies an altered protein secretion pattern due to *podB1* mutation. (**A**) Abundance of proteins secreted in ASH83@RT compared to A28@PT. 747 proteins were identified in the secretomes of both A28@RT and ASH83@RT. In comparison of ASH83@RT and A28@PT, 67 were found in lower abundance while 517 were found in higher abundance in the secretome (**B**) GO – analysis based on biological function of proteins secreted in lower abundance in ASH83@RT compared to A28@RT. (**C**) GO – analysis based on cellular location of proteins secreted in higher abundance in ASH83@RT compared to A28@RT. Abbreviations; ER – Endoplasmic Reticulum and PM – Plasma Membrane.

To evaluate the effect of *podB1* mutation on the types of proteins secreted, we performed a quantitative proteomic analysis of culture filtrates. Both A28 and ASH83 strains were initially grown in minimal medial (MM), for 18 h, at 28 °C. They were then shifted to fresh MM and grown for 6 h at 42 °C, allowing the *podB1* phenotype to manifest. Secreted proteins from both cultures were examined using a mass spectrometry based approach to carry out a proteomic analysis (Supplementary Fig. 3). We identified 747 proteins (Supplementary Table S5), in both biological replicates of both strains. While there were no differences in secretion for 163 proteins, 517 proteins were secreted in higher abundance (≥1.5 fold), and 67 proteins were secreted in lower abundance (≤0.67) in ASH83 compared to A28 at the restrictive temperature (Fig. 2A).

To further characterize these proteins, they were clustered using GO annotations assigned to biological function and components in fungi. Of the proteins secreted in lower abundance in ASH83, 50% were classified as cell wall related proteins, 20% as hydrolytic proteins and 30% were related to other biological functions (Fig. 2B). Of the proteins secreted in higher abundance, 16% were classified as extracellular proteins, 8% as plasma membrane proteins and 6% as cell wall proteins. 11% proteins could not be grouped while the rest of the proteins were categorized as intracellular proteins (Fig. 2C). Upon categorizing the proteins based on biological function, we observed that the majority of the proteins (~60%) were components of the protein secretion machinery, while 25% were classified as hydrolytic proteins. The other 15% of proteins could not be grouped as their functions are still unknown.

### The UPR is up regulated in ASH83 at restrictive temperature

**Figure 3:**
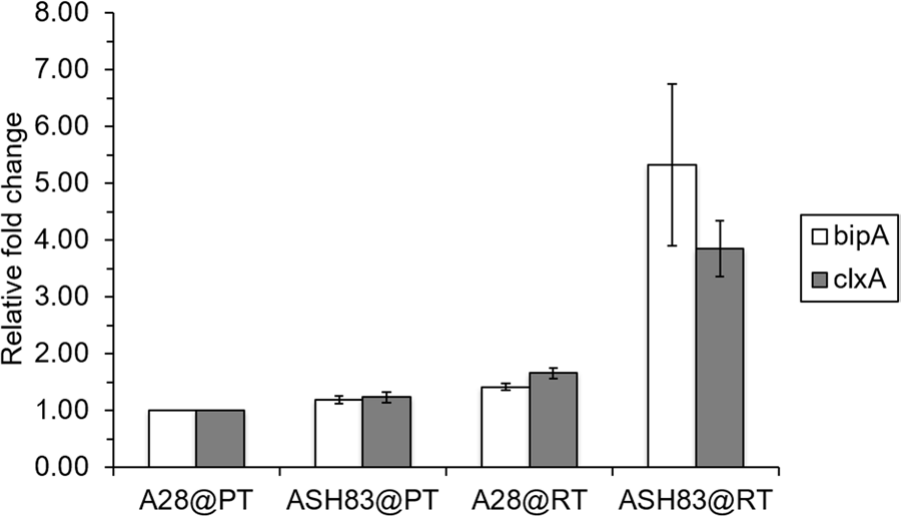
Analysis of the expression of genes involved in UPR. Fold change of *bipA* and *clxA* gene expression relative to A28@PT in both A28 and ASH83 at both 28 °C and 42 °C. Actin was used as internal control for each qPCR reaction. Data is representative of three biological replicates and three technical replicates.

In the analysis of proteins secreted in higher abundance in ASH83 compared to A28 at the restrictive temperature, we observed the presence of chaperones, foldases and other downstream targets of UPR. Accordingly, we hypothesized that the UPR is upregulated in ASH83 at the restrictive temperature. To test this hypothesis, we performed transcriptional analysis of UPR genes using qRT-PCR analysis. Similar to the conditions used for secretome proteomics, A28 and ASH83 were initially grown in MM for 18 h at 28 °C. Media was then replaced and strains were grown at 6 h at either permissive or the restrictive temperature before isolating RNA. We analyzed transcript levels of the UPR related genes *bipA*^24^ and *clxA*^25^ to assess the induction of UPR, as it was previously reported that *bipA* is a putative UPR gene in *A. nidulans*^24^ (Fig. 3). In ASH83 grown at 42 °C, levels of both *bipA* and *clxA* transcripts are significantly higher than in controls, implying that the *podB1* mutation triggers UPR activation.

### Effect of the *podB1* mutation on cell wall organization

**Figure 4:**
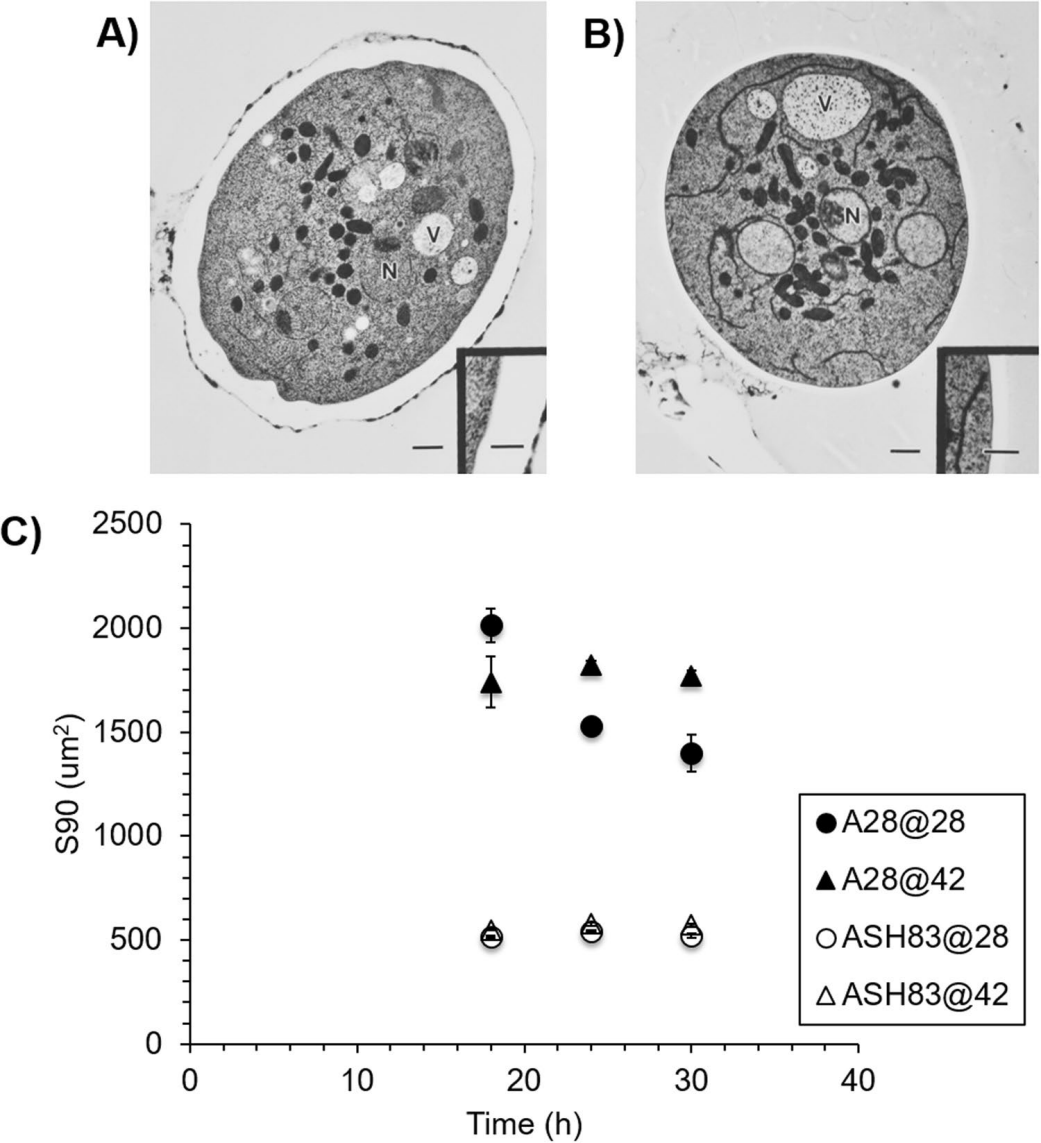
Effect of podB1 mutation on hyphal cell wall. (**A**) TEM images of ASH83 grown at 28 °C and (**B**) 42 °C. Insets: enlargement of cell wall. Scale bar = 1μm or 0.5μm (insets) N marks nuclei; V marks vacuoles. (**C**) The average size of the fungal elements in the liquid culture. *Y* - axis is representative of the average size under 90-percentile level. Mutation in podB affects the size of fungal elements at both 28 °C and 42 °C. Data is representative of 3 biological replicates.

To assess the impact of the *podB1* mutation on cell wall structure, ASH83 was grown at 42 °C, fixed by plunge freezing and freeze substitution, and examined using transmission electron microscopy. Most of the ASH83 cells grown at 42 °C collapsed during the fixation process. It seems likely they were weakened by the large number of vacuoles present or by the wall abnormalities. In the surviving ASH83 cells grown at 42 °C, the wall was approximately four times thicker and significantly less regular than ASH83 cells grown at 28 °C (Fig. 4A,B). To further characterize the effect of *podB1* on cell walls, we evaluated the average size of mycelia during shake flask growth. Both A28 and ASH83 strains were grown at the permissive temperature for 18 hours before shifting to the restrictive temperature. We observed that the average size of the mycelia for ASH83 were significantly smaller than the control strain grown at at both permissive and the restrictive temperatures (Fig. 4C). This implies that ASH83 hyphae possess weaker cell walls that fragment during growth, resulting in smaller hyphae. We note this was the only phenotype in which we observed the effect of the *podB1* mutation at the permissive temperature (28 °C).

### Proteomic changes in the cell wall due to the *podB1* mutation

**Figure 5:**
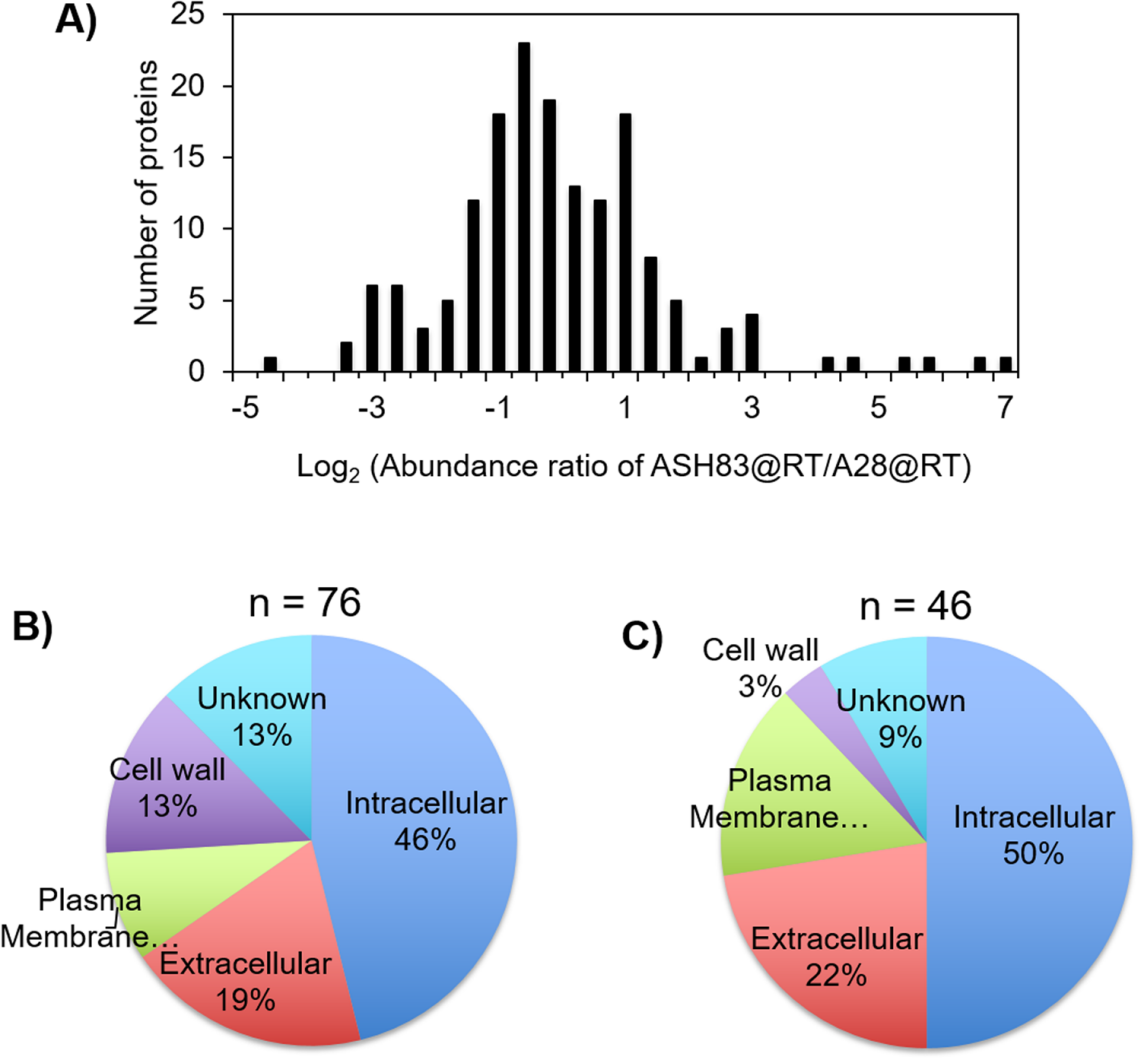
Quantitative cell wall proteomic analysis identifies differences in proteins expressed in cell wall of ASH83. (**A**) Abundance of proteins found in ASH83@RT compared to A28@RT. 165 proteins were identified in the cell wall components of A28 and ASH83. 46 proteins were expressed in higher abundance while 76 proteins were expressed in lower abundance in ASH83@RT compared to A28@RT. (**B**) GO – analysis based on cellular component of cell wall proteins expressed in lower abundance in ASH83@RT compared to A28@RT. (**C**) GO – analysis based on cellular component of cell wall proteins expressed in higher abundance in ASH83@RT compared to A28@RT.

To further understand the basis for the weakened cell wall of ASH83, we characterized its cell wall proteome relative to that of wild type strains. In order to appropriately capture the proteomic effects of the *podB1* mutation on the cell wall, we grew ASH83 only at 42 °C for 16 h before isolating the cell walls. We also grew ASH83 at 28 °C for 16 h, as an isogenic control strain. But to account for changes in phenotype associated at growth at 42 °C, we also grew A28 at both 28 °C and 42 °C. Proteins isolated from the strains were examined by LC-MS/MS based proteomic analyses (Supplementary Fig. 4). We were able to identify 165 proteins (Supplementary Table S6) that were common to both the replicates (Fig. 5A). Out of these 165 proteins, 46 proteins were expressed in higher abundance while 76 proteins were found to have lower abundance in ASH83 compared to A28 grown at the restrictive temperature.

Bioinformatic analysis of proteins was carried out to group the proteins using GO slim mapper based on cellular components. We observed that the low abundant proteins were classified based on cellular location into five groups, i.e. intracellular (46%), extracellular (19%), plasma membrane (9%), cell wall (13%) and unknown (13%) (Fig. 5B). Similarly, the high abundant proteins were grouped into five groups viz., intracellular (50%), extracellular (22%), plasma membrane (16%), cell wall (3%) and unknown (9%) (Fig. 5C). The presence of large number of intra cellular proteins is unusual but is similar to previous reports of the presence of intracellular proteins in cell wall proteome^22^.

## Discussion

While much is known regarding the eukaryotic protein-secretory pathway, many details related to protein secretion in the filamentous fungi are not. However, it has been demonstrated that increases in protein secretion are achieved in strains with mutations affecting protein or vesicle trafficking and glycosylation^14,21^. Both vesicle trafficking and glycosylation appear to be impacted by PodB. Of interest here, PodB was recently shown to be a homolog of COG2, a member of the multi-subunit Conserved Oligomeric Golgi (COG) tethering complex which mediates vesicle trafficking within the Golgi apparatus^15^. In *S. cerevisiae*, COG2 is involved in membrane trafficking and protein transport^17,18^. In *A. nidulans*, PodB plays a role in polarity development, germination, and protein glycosylation^15^. Yet, up to now, its role in protein secretion has been unclear in filamentous fungi. Accordingly, in this study, we sought to gain a better understanding of the global impacts of the *podB1* mutation on protein secretion. To do this, we characterized the phenotypic effects of *A. nidulans podB1* mutation on growth and morphology, and evaluated the mutation-mediated, protein expression changes in both the secretome and hyphal cell walls.

Polarity establishment and polarity maintenance are fundamental processes, critical for appropriate hyphal morphogenesis in filamentous fungi^26^. At the restrictive temperature, the *podB1* mutation manifested and led to isotropic growth, swelling of spores, and lack of germination (Supplementary Fig. S1D). Similar growth defects have been observed at the restrictive temperature in Ts mutants of *swoC, swoD and swoF*^27^, implying that PodB is involved in A. *nidulans* polarity establishment, corroborating previous work^19^. During shake-flask growth at the permissive temperature (28 °C), the specific growth-rate of ASH83 was similar to that of the control strain. However, after a shift to the restrictive temperature, the control strain continued to grow while the ASH83 stopped producing additional biomass (Fig. 1A). This arrest in growth of biomass at the restrictive temperature was similar to that observed in *S. cerevisiae* mutants with temperature sensitive mutations in *COG2*^18^. These data suggest that PodB is required for filamentous growth in *A. nidulans*.

In addition to the impact the *podB1* mutation has on growth, we observed the specific productivity of ASH83 to be 15 times higher than that of a control strain at the restrictive temperature. These data are consistent with previous reports that mutation of *arl1*, a bonafide GTPase that colocalizes in the Golgi, leads to increased protein secretion in *C. albicans*. Similar to PodB, Arl1 is required for polarized growth in *C. ablicans* and the increase in protein secretion due to *arl1/arl1* mutation appears to be due to unregulated protein secretion^28^. Furthermore, loss of function of PodB results in changes in post-translational modifications in *A. nidulans*^15^. However, these changes in post-translational modifications did not reduce the activity of cellulases or xylanases in ASH83 at restrictive temperature.

An increase in specific protein productivity in ASH83 at restrictive temperature is similar to previous reports of increases in protein secretion observed in A. oryzae mutants defective in trafficking proteins such as Vps10^29^, Emp47 and Vip36^14^. It has also been shown that the widely used Trichoderma reesei RutC-30 cellulase hyper-production strain possesses mutations of trafficking proteins including Vps13, Golgi associated vps1 vacuolar ATPase, and the Cog1 subunit of the COG complex^30^. COG2 has also been previously reported to be involved in the double membrane formation in the autophagy pathway in *S.cerevisiae*^31^ and deletion of autophagy genes resulted in a three-fold increase in secreted bovine chymosin in *A. oryzae*^32^. In addition, a previous report^33^ has shown Aspergillus autophagosomes form in close association with ring-shaped ER structures, which represents a potential link between ER stress and autophagy. These data suggest mutation of trafficking proteins to create alternative secretory profiles of both homologous and heterologous proteins in fungi may be an attractive strategy for design of filamentous fungal strains.

Comparing the secretomes of a ASH83 and A28 at the restrictive temperature, we found that ASH83 showed an increase in secretion of a wide variety of intracellular proteins. To determine if the *podB1* mutation leads to cellular lysis at restrictive temperature, we assessed the integrity of ASH83. Fungi were simultaneously stained with Calcafluor white (to image cell walls) and NUCLEAR-ID Red (to image nuclear DNA) to assess the integrity of ASH83. If the *podB1* mutation causes ASH83 to lyse at restrictive temperature, subcellular components including nuclei will be released into the extracellular medium. However, if nuclei are found within mycelia, it suggests ASH83 remains intact when grown at restrictive temperature. Out of n = 40 hyphal tip cells photographed 100% contained nuclei, indicating that these mutant cells remained intact after the shift to restrictive temperature. These data suggest the *podB1* mutation does not lead to cell lysis in ASH83. Additionally, Gremillion *et al*.^15^ have shown ASH83 exhibits isotropic growth at 42 °C, but then germinates after a shift from restrictive to permissive temperature. This suggests the effect of the *podB1* mutation can be reversed by shifting the temperature and that viability of ASH83 remains intact even when grown at restrictive temperature for a prolonged period of time.

Furthermore, we observed that most of the proteins secreted in higher abundances in ASH83 at restrictive temperature were intracellular proteins including: ribosomal proteins, chaperones, foldases and others involved in the protein synthetic machinery. As these proteins represent gene products involved in the UPR^34^, we sought to determine if characteristic UPR genes were up regulated in ASH83 at the restrictive temperature. We found increased expression of the UPR genes, *bipA* and *clxA*, only when ASH83 was grown at the restrictive temperature, suggesting that the UPR is induced by the *podB1* mutation (Fig. 4). We speculate this induction in UPR may occur for several possible reasons: (a) the *podB1* mutation directly induces the UPR, (b) differences in glycosylation caused by the *podB1* mutation may cause UPR induction as reported earlier^35^ or (c) loss of PodB function affects overall Golgi physiology. These changes in Golgi physiology may alter Golgi function; which would increase the stresses in the ER thus leading to induction of the UPR. *BipA*, and *clxA*, the chief components of UPR^25,36^ are known to be involved in protein folding, relieving the stresses of protein overproduction in the ER^37^. Previous reports regarding increased secretion of homologous proteins, such as α-amylase in *S. cerevisiae*^38^ and heterologous proteins such as laccase in *T. versicolor* and bovine preprochymosin in *A. niger*^13^, suggest activation of UPR may, in part, be responsible for increases in protein secretion observed in ASH83 at the restrictive temperature.

Previous studies have described morphological defects caused by the *podB1* mutation^15,19^. We have shown here that this mutation causes additional defect consistent with a role for PodB in membrane trafficking. These include the presence of a large number of vacuoles and much less prominent endomembranes. Notably, the former phenotype has also been observed in *S. cerevisiae* mutants that are defective in ER to Golgi transport^18^. Nevertheless, our TEM observations reveal that hyphal tips are relatively normal in hyphae that have been upshifted for a short time period (Supplementary Fig. S5).

Additionally, cell wall structure also appears to be affected by the *podB1* mutation. The cell wall of ASH83 at the restrictive temperature was about four times thicker and significantly less regular than the ASH83 cell wall at the permissive temperature. This finding is consistent with previous reports that *podB1* mutation affects cell wall morphology^15^. Using quantitative proteomic analysis, we found the ASH83 cell wall was drastically altered at the restrictive temperature. We observed the presence of a large number of intracellular proteins in the cell walls of both A28 and ASH83 at both permissive and the restrictive temperatures, consistent with earlier reports regarding the presence of nominally intracellular proteins in yeast cell walls^22^. We observed that most of the canonical cell wall proteins (CWP’s) were found in lower abundance in ASH83 compared to A28 during growth at the restrictive temperature. As cell wall protein composition is a direct result of protein secretory pathway in *A. nidulans*, we speculate that the loss of function of PodB disrupts the balance between endocytosis and exocytosis similar to loss of function of other Golgi proteins such as RabO, SedV^39^ and HypB^40^. Hence, this loss of balance leads to lower abundances of cell wall proteins in ASH83 at restrictive temperature.

In ASH83, we found that hydrophobins were the only canonical fungal CWP’s found in higher abundance in ASH83 compared to A28 at restrictive temperature. We presume this was due to the isotropic growth observed in ASH83 at the restrictive temperature. Furthermore, upon comparing the proteins identified in the cell wall fraction to those in the secretome of ASH83, we found that approximately 70% of cell wall associated proteins to be secreted in higher abundances in ASH83 compared to A28 at the restrictive temperature. These data, combined with the fact that hyphae of ASH83 used for cell wall proteomics had no polar growth (Supplementary Fig. S1D), suggests that protein secretion in the absence of PodB relies upon an unconventional pathway that differs from the normal vectorial ER to Golgi route^37^. Additionally, since the cell wall is the last barrier for secretion, we speculate that the weakened cell wall phenotype may somehow facilitate this unconventional process.

Furthermore, we discovered that the average size of hyphae of ASH83, during growth at both 28 °C and 42 °C, were considerably smaller than A28 during growth at the same temperatures (Fig. 5C). The smaller size of the ASH83 mycelia suggests that ASH83 fragments more easily than A28 during flask growth, thus resulting in smaller hyphae. Fragmentation of fungi into smaller hyphae in the fermentation process is beneficial, as it has been previously reported that hyphae with smaller projected area lead to lower viscosity broth. As a result, an increase in levels of oxygen mass transfer and improved productivity has been observed^41^.

Together, these results suggest that strategies for the design and engineering of industrial fungal strains that maximize secretion of desired proteins should include mutations that affect COG function. Indeed, it has already been shown that the widely used RutC-30 cellulase hyper-production strain of the fungus *Trichoderma reesii* possesses a mutation affecting the Cog1 subunit of the COG complex^30^. How this and the *podB1* mutation might contribute in enhancing the productivity remains unknown. Possible explanations include: (i) altered glycosylation of secreted proteins, (ii) weakening of the cell wall, (iii) improved folding of secreted proteins due to activation of the UPR, and/or (iv) an increase in the surface area available for secretion due to loss of hyphal polarity.

## Materials and Methods

### *Aspergillus* strains and growth media

All the chemicals used in this study were obtained from Sigma-Aldrich, MO, USA unless specified. Media used for the study was the standard media that has been used previously to study different phenomena in filamentous fungi^42–44^. The media used was MAG (2% malt extract, 0.2% peptone (BD-Difco, NJ, USA), 1% dextrose (Fisher Scientific, MA, USA), 0.01% vitamin mix and trace elements)^42^, YGV (0.5% yeast extract (Fisher Scientific, MA, USA), 2% dextrose, 0.01% vitamin mix and trace elements), CM (1% dextrose, 0.2% peptone, 0.1% yeast extract, 0.1% casamino acids, 0.01% vitamin mix, nitrate salts, and trace elements) or MM (1% carbon source (glucose or starch or carboxymethyl cellulose (CMC)), nitrate salts, 0.01% vitamin mix and trace elements). When necessary, 2% agar was added to solidify media. Details vitamin mix, trace elements mix and nitrate salts are described in Supplementary Tables S1–S3 respectively. The strains used in this study are FGSC A28 (*pabaA6 biA1*; obtained from the Fungal Genetics Stock Center (Manhattan, KS) and ASH83 (*pabaA6 podB1 pyroA4*; Harris *et al*., 1999).

### Cellulase and Xylanase enzyme assays

Mycelia were grown in MM with CMC as the carbon source for assaying the activities of cellulases and xylanases. Cellulase activity was measured by colorimetric assay using Avicel as substrate. The reducing sugars were determined according to the Miller procedure^45^ using glucose as control. The reaction mixture (0.05 mL substrate (1% w/v) in 50 mM sodium acetate buffer, pH 5.0, and 0.05 mL enzyme solution) was incubated at 50 °C for 60 min. The reaction was stopped by adding 0.1 mL of DNS and boiling immediately for 5 min. Xylanolytic activity was measured using birchwood xylan as substrate, and the reducing sugars were determined according to the Miller procedure^45^. Quantification of the reducing sugars released as a result of enzyme activity was estimated by A_540_ measurements, where one unit of enzymatic activity was defined as the amount of enzyme that produced 1 μmol.min^−1^ of reducing sugars.

### Microscopic imaging of hyphae

For coverslip inoculation using strains, 100,000 spores were pipetted onto coverslips pretreated with Concanavalin A (Sigma-Aldrich, MO, USA). Concanavalin A is a lectin that specifically binds to carbohydrates such as mannose and glucose^46^. It is commonly used to immobilize yeast cells for live cell imaging^47^. Coverslips with spores were initially incubated in MM for 14 h at 28 °C before they were growth in fresh MM for 6 h at either 28 °C or 42 °C. The coverslips were then stained with NUCLEAR-ID Red DNA (Enzo life sciences, NY, USA) and Calcofluor White (Sigma-Aldrich, MO, USA). Stained mycelia on the coverslips were then imaged using Zeiss Axiometric 200 microscope (Carl Zeiss Werk, Gottinger, Germany).

### Transmission electron microscope (TEM)

Conidia were inoculated onto a single, moist layer of sterile dialysis tubing, pre-cut into 3 mm square pieces, and placed on supplemented complete solid medium. Conidia were incubated at either 30 °C or 42 °C for 17 hours, or at 30 °C for 11 hours followed by 42 °C for 5 hours. After incubation, dialysis squares were removed from plates and immediately plunge frozen in liquid propane according to the procedure of Harvey^48^, and then transferred to vials of substitution fluid as described by Mims *et al*.^49^. After 4 days at −80 °C, the vials were warmed slowly to room temperature and rinsed with three changes of HPLC grade acetone. Infiltration with Araldite, Embed 812 (Epon-812) resin was carried out at room temperature as follows: 1 part resin to 2 parts acetone overnight; 2 parts resin to one part acetone for 8 hours; 3 changes of 100% resin for 8–12 hours each. Samples were flat embedded between Permanox slides and polymerized for 48 hours at 60 °C. Samples were sectioned with an RMC 6000XL Ultramicrotome equipped with a diamond knife. Sections were picked up on slot grids, allowed to dry on Formvar-coated racks^50^ and post stained for 3 minutes in aqueous uranyl acetate followed by lead citrate^51^. Grids were examined in a Zeiss EM 902 A TEM operated at 80 kV.

### Isolation of cell walls

Cell wall proteins (CWP’s) were isolated as previously reported with minor modifications^52^. Mycelia were crushed using 1 mm beads in a Precellys 24 homogenizer (Bertin instruments, France). Cytoplasmic proteins and other non-cell wall components were removed by washing the crushed mycelia in 10 mM Tris (pH 7.5). Cell wall components were then washed extensively in 1 M NaCl solution containing protease inhibitor to remove ions associated with cell walls. The cell walls were then boiled in reducing buffer (50 mM Tris pH 8.0, 0.1 M EDTA, 10 mM DTT) to remove proteins covalently bound by disulfide bonds to CWP’s and any other residual cytosolic components. Cell walls were then washed with water 6 times to remove residual reducing buffer. Washed cell walls were lyophilized before extracting the CWP’s.

### Extraction of CWP

Major components of fungal cell walls such as GPI-anchored proteins and alkali proteins were isolated using published methods^52^. GPI-anchored proteins were isolated by treating 20 mg of lyophylized cell wall mass with 300 µl of hydrogen fluoride pyridine at 4 °C for 3 h. The reaction was stopped using equal amounts of ice-cold water. Alkali proteins (e.g., Pir proteins) were extracted by treating 1 g of wet cell mass with 4 ml of 30 mM NaOH at 4 °C for 17 h. The reaction was quenched by neutralizing the NaOH with acetic acid. All the extracted proteins were then pooled together to quantify the amount of proteins by BCA assay.

### Sample preparation for mass spectrometry

Filter aided sample preparation (FASP) was used for mass spectrometry sample preparations with minor modifications^53^. Briefly, up to 100 µg of proteins were reduced in 7.5 mM Tris(2-carboxyethyl)phosphine hydrochloride (TCEP-HCL) (Thermo Scientific, MA, USA) at 37 °C for 1 h. TCEP-HCL was filtered out of the protein mixture by using 3 K MWCO filters (EMD Millipore, MA, USA). Proteins were then alkylated using 50 mM Iodoacetamide at room temperature for 1 h in the dark. The alkylation reaction was stopped by using 25 µl of 500 mM DTT. The iodoacetamide/DTT mixture was then filtered out from the protein mixture by using 3 K MWCO filters. Proteins were washed repeatedly with 50 mM sodium bicarbonate before digesting with trypsin at 50:1 (W/W) ratio at 37 °C overnight.

### Tandem Mass Tag (TMT) labeling

Peptides digested by Trypsin were labeled with TMT tags (TMTsixplex kit, Thermo Scientific, MA, USA) as per vendor instructions. Briefly, isobaric mass tags were brought to room temperature and resuspended in 41 µl of anhydrous acetonitrile. Up to 100 µg of peptides were then added to the isobaric mass tag and incubated at RT for 1 h. The tagging reaction was quenched by adding 8 µl of 5% hydroxylamine for 15 mins at RT. Unbound tags were removed from the tagged samples using C18 columns before mass spectrometric analysis. Tagged peptides were quantified using a calorimetric peptide assay (Thermo Scientific, MA, USA) according to manufacturers instructions. Peptides from all the samples were mixed 1:1:1:1 (W/W) ratio before mass spectrometric analysis.

### LC-MS/MS analysis

NanoLC-MSMS analyses were carried out using a UltiMate™ 3000 RSLCnano system interfaced to an orbitrap Fusion Lumos tribrid mass spectrometer (Thermo Scientific, San Jose, CA). 1ug of tagged peptides were loaded into an Accalaim PepMap™ 100(5 µm, 100 Å, 300 µm × 5 mm) trap column at 5 µL/min with 100% solvent A (2.5% ACN, 0.1% formic acid) for 5 min, then eluted and separated with an Accalaim PepMap™ 100 nano column (3 µm, 100 Å, 75 µm × 250 mm) with a linear gradient of 2–50% solvent B (75% ACN, 0.1% formic acid) over 160 min.

Precursor masses were detected in the orbitrap at R = 120,000 (m/z 200). Data dependent MS2 and MS3 were carried with top of speed setting, cycle time is 3 sec. Full scan MS1 spectra were recorded in the range of m/z 400–1600 in the orbitrap at R = 120,000 (m/z 200). For MS2, peptides were fragmented using collision induced dissociation (CID) and analyzed using the linear ion trap. The 10 most abundant fragments in each MS2 spectrum were isolated with “synchronized precursor isolation” and fragmented using high-energy collision induced dissociated (HCD). Reported ions from MS3 were recorded in the orbitrap.

### Mass spectrometric data analysis

MS data were analyzed using Proteome Discoverer 2.1. (Thermo Scientific, San Jose, CA) Spectra were searched using the Sequest HT (Thermo Scientific, San Jose, CA) engine against an *Aspergillus nidulans* protein database containing 10555 entries downloaded from Uniprot. Trypsin was chosen as digestion enzyme with up to two missed cleavages. Fixed modifications included carbamidomethylation of cysteines and TMT modification of peptide N-terminus and Lysine residues. Oxidation of methionines was set as a variable modification. Peptide spectral matches (PSM) were filtered to a 1% FDR using the Percolator algorithm. The TMTsixplex quantification method was used to calculate the reporter ion ratios. Only the peptide spectra containing the reporter ions with an intensity of over 10 units were used for analysis as suggested by the manufacturer. Protein ratio is expressed as a median of the ratios of all peptides quantified. Both the secretome and cell wall proteomic analyses were performed in duplicates and the changes in abundance ratio reported are the mean of the replicates.

### Quantitative reverse transcription – PCR (qRT-PCR)

ASH83 and A28 strains were grown in MM at 28 °C for 18 h before replacing with fresh MM. Strains were then grown at either 28 °C or 42 °C for 6 h. RNA was isolated from these strains using RNeasy Plus Universal Mini Kit (Qiagen, MD, USA) according to the instructions provided by the manufacturer. The quality of mRNA was verified by running on agarose gels before further processing. 500 ng of mRNA was reverse transcribed into cDNA using the High-Capacity cDNA Reverse Transcription Kit (Thermo fisher Scientific, MA, USA) according to manufacturer’s instructions. qRT-PCR was performed by quantifying the amplification of cDNA with the CFX96 Real Time system (Bio-rad, CA, USA) and SYBY Green PCR Master Mix (Thermo fisher, MA, USA). For all the genes tested, we performed three biological replicates and three technical replicates. Actin was used as the internal control. Primers used for amplification are detailed in Supplementary Table S4. After PCR, melting curves were established for all the reactions in the range of 60–95 °C to evaluate the specificity of amplification. All data was analyzed using the ΔΔCT method^54^. A28 grown at 28 °C was used as the control strain for the analysis. Statistical analysis was performed in Excel (Microsoft, WA, USA).

### Particle size analysis

Fungal elements were measured using a Malvern Mastersizer 3000 (Malvern Instruments, UK) system as reported earlier^55^. Briefly, Strains were grown at 28 °C for 18 h (until the biomass reached at least 1 g/Kg), before shifting the temperature to 42 °C for the strains that have to be grown at the restrictive temperature. For each time point where the sizes of fungal elements were measured, 1 ml of the sample was dispersed in tap water for analysis in the Malvern Mastersizer 3000. Tap water was used to minimize bubbles in the measuring prism. Stirring was set at 1800RPM and at least 1% laser saturation was used for stable reading. The 90^th^ percentile of the particle size distribution, S_90_ was used as the parameter to measure the average size of fungal element.

### Data availability

All the data generated or analyzed during the current study are included in this published article and its supplementary information files.

## Electronic supplementary material

Supplementary information

Supplementary table S5

Supplementary table s6

## References

[1] Cherry JR, Fidantsef AL (2003). Directed evolution of industrial enzymes: an update. Curr Opin Biotechnol.

[2] Gupta, V. K., Mach, R. L. & Sreenivasaprasad, S. Fungal biomolecules: sources, applications, and recent developments. (John Wiley &amp; Sons, Inc., 2015).

[3] Punt, P. J. *et al*. Filamentous fungi as cell factories for heterologous protein production. *Trends Biotechnol* **20**, 200–206, S0167-7799(02)01933-9 (2002). 10.1016/s0167-7799(02)01933-9 11943375

[4] Yoon J, Maruyama J, Kitamoto K (2011). Disruption of ten protease genes in the filamentous fungus Aspergillus oryzae highly improves production of heterologous proteins. Appl Microbiol Biotechnol.

[5] Liu T, Wang T, Li X, Liu X (2008). Improved heterologous gene expression in Trichoderma reesei by cellobiohydrolase I gene (cbh1) promoter optimization. Acta Biochim Biophys Sin (Shanghai).

[6] Gouka RJ, Punt PJ, van den Hondel CA (1997). Glucoamylase gene fusions alleviate limitations for protein production in Aspergillus awamori at the transcriptional and (post) translational levels. Appl Environ Microbiol.

[7] Wang L, Ridgway D, Gu T, Moo-Young M (2005). Bioprocessing strategies to improve heterologous protein production in filamentous fungal fermentations. Biotechnol Adv.

[8] Hong F, Meinander NQ, Jönsson LJ (2002). Fermentation strategies for improved heterologous expression of laccase in Pichia pastoris. Biotechnol Bioeng.

[9] Nevalainen H, Peterson R (2014). Making recombinant proteins in filamentous fungi- are we expecting too much?. Front Microbiol.

[10] Lodish, H. F. Molecular cell biology. 7th edn, (W.H. Freeman and Co., 2013).

[11] Schröder M, Kaufman RJ (2005). The mammalian unfolded protein response. Annu Rev Biochem.

[12] Malavazi I, Goldman GH, Brown NA (2014). The importance of connections between the cell wall integrity pathway and the unfolded protein response in filamentous fungi. Brief Funct Genomics.

[13] Valkonen M, Ward M, Wang H, Penttilä M, Saloheimo M (2003). Improvement of foreign-protein production in Aspergillus niger var. awamori by constitutive induction of the unfolded-protein response. Appl Environ Microbiol.

[14] Hoang HD, Maruyama J, Kitamoto K (2015). Modulating endoplasmic reticulum-Golgi cargo receptors for improving secretion of carrier-fused heterologous proteins in the filamentous fungus Aspergillus oryzae. Appl Environ Microbiol.

[15] Gremillion SK (2014). Mutations in proteins of the Conserved Oligomeric Golgi Complex affect polarity, cell wall structure, and glycosylation in the filamentous fungus Aspergillus nidulans. Fungal Genet Biol.

[16] Suvorova ES, Duden R, Lupashin VV (2002). The Sec. 34/Sec. 35p complex, a Ypt1p effector required for retrograde intra-Golgi trafficking, interacts with Golgi SNAREs and COPI vesicle coat proteins. J Cell Biol.

[17] Ram RJ, Li B, Kaiser CA (2002). Identification of Sec. 36p, Sec. 37p, and Sec. 38p: components of yeast complex that contains Sec. 34p and Sec. 35p. Mol Biol Cell.

[18] Wuestehube LJ (1996). New mutants of Saccharomyces cerevisiae affected in the transport of proteins from the endoplasmic reticulum to the Golgi complex. Genetics.

[19] Harris SD, Hofmann AF, Tedford HW, Lee MP (1999). Identification and characterization of genes required for hyphal morphogenesis in the filamentous fungus Aspergillus nidulans. Genetics.

[20] Helenius A, Aebi M (2001). Intracellular functions of N-linked glycans. Science.

[21] Dai Z (2013). Impact of alg3 gene deletion on growth, development, pigment production, protein secretion, and functions of recombinant Trichoderma reesei cellobiohydrolases in Aspergillus niger. Fungal Genet Biol.

[22] Bowman SM, Free SJ (2006). The structure and synthesis of the fungal cell wall. Bioessays.

[23] Takuji, O., Taiki, F. & Masatoshi, G. In Stress Biology of Yeasts and Fungi (eds Takagi Hiroshi & Kitagaki Hiroshi) 151–168 (Springer Japan, 2015).

[24] Saloheimo M, Valkonen M, Penttilä M (2003). Activation mechanisms of the HAC1-mediated unfolded protein response in filamentous fungi. Mol Microbiol.

[25] Wang H (2003). Isolation and characterisation of a calnexin homologue, clxA, from Aspergillus niger. Mol Genet Genomics.

[26] Steinberg, G., Peñalva, M. A., Riquelme, M., Wösten, H. A. & Harris, S. D. Cell Biology of HyphalGrowth. Microbiol Spectr **5**, 10.1128/microbiolspec.FUNK-0034-2016 (2017). 10.1128/microbiolspec.funk-0034-2016 PMC11687463 28429675

[27] Momany M, Westfall PJ, Abramowsky G (1999). Aspergillus nidulans swo mutants show defects in polarity establishment, polarity maintenance and hyphal morphogenesis. Genetics.

[28] Labbaoui H (2017). Role of Arf GTPases in fungal morphogenesis and virulence. PLoS Pathog.

[29] Yoon J, Aishan T, Maruyama J, Kitamoto K (2010). Enhanced production and secretion of heterologous proteins by the filamentous fungus Aspergillus oryzae via disruption of vacuolar protein sorting receptor gene Aovps10. Appl Environ Microbiol.

[30] Le Crom S (2009). Tracking the roots of cellulase hyperproduction by the fungus Trichoderma reesei using massively parallel DNA sequencing. Proc Natl Acad Sci USA.

[31] Yen WL (2010). The conserved oligomeric Golgi complex is involved in double-membrane vesicle formation during autophagy. J Cell Biol.

[32] Yoon J, Kikuma T, Maruyama J, Kitamoto K (2013). Enhanced production of bovine chymosin by autophagy deficiency in the filamentous fungus Aspergillus oryzae. PLoS One.

[33] Pinar M, Pantazopoulou A, Peñalva MA (2013). Live-cell imaging of Aspergillus nidulans autophagy: RAB1 dependence, Golgi independence and ER involvement. Autophagy.

[34] Travers KJ (2000). Functional and genomic analyses reveal an essential coordination between the unfolded protein response and ER-associated degradation. Cell.

[35] Gerlach, J. Q., Shashank, S. & Lokesh, L. K. J. J. In Endoplasmic Reticulum Stress in Health and Disease (eds Agostinis Patrizia & Afshin Samali) 23–39 (Springer Netherlands, 2012).

[36] Bertolotti A, Zhang Y, Hendershot LM, Harding HP, Ron D (2000). Dynamic interaction of BiP and ER stress transducers in the unfolded-protein response. Nat Cell Biol.

[37] Conesa A, Punt PJ, van Luijk N, van den Hondel CA (2001). The secretion pathway in filamentous fungi: a biotechnological view. Fungal Genet Biol.

[38] Valkonen M, Penttilä M, Saloheimo M (2003). Effects of inactivation and constitutive expression of the unfolded- protein response pathway on protein production in the yeast Saccharomyces cerevisiae. Appl Environ Microbiol.

[39] Pinar M, Pantazopoulou A, Arst HN, Peñalva MA (2013). Acute inactivation of the Aspergillus nidulans Golgi membrane fusion machinery: correlation of apical extension arrest and tip swelling with cisternal disorganization. Mol Microbiol.

[40] Yang Y, El-Ganiny AM, Bray GE, Sanders DA, Kaminskyj SG (2008). Aspergillus nidulans hypB encodes a Sec. 7-domain protein important for hyphal morphogenesis. Fungal Genet Biol.

[41] Bhargava S, Wenger KS, Rane K, Rising V (2005). & Marten, M. R. Effect of cycle time on fungal morphology, broth rheology, and recombinant enzyme productivity during pulsed addition of limiting carbon source. Biotechnol Bioeng.

[42] W., H. T. & Etta, K. Improved protocols for Aspergillus minimal medium: trace elements and minimal medium salt stock solutions, http://www.fgsc.net/fgn48/Hillfinal.pdf (2001).

[43] Dynesen J, Nielsen J (2003). Branching is coordinated with mitosis in growing hyphae of Aspergillus nidulans. Fungal Genet Biol.

[44] Si H, Justa-Schuch D, Seiler S, Harris SD (2010). Regulation of septum formation by the Bud3-Rho4 GTPase module in Aspergillus nidulans. Genetics.

[45] Miller GL (1959). Use of Dinitrosalicylic Acid Reagent for Determination of Reducing Sugar. Analytical Chemistry.

[46] Goldstein, I. J. & Poretz, R. D. In *The Lectins: Properties, Functions, and Applications in Biology and Medicine* (ed. Liener Irvin) 33–247 (Elsevier, 2012).

[47] Pemberton LF (2014). Preparation of yeast cells for live-cell imaging and indirect immunofluorescence. Methods Mol Biol.

[48] Harvey, H. C. In Ultrastructure Techniques for Microorganisms (eds Aldrich Henry, C. & Todd William, J.) 183–212 (Springer US, 1986).

[49] Mims CW, Richardson EA, Timberlake WE (1998). Ultrastructural analysis of conidiophore development in the fungus *Aspergillus nidulans* using freeze-substitutionUltrastructural analysis of conidiophore development in the fungus Aspergillus nidulans using freeze-substitution. Protoplasma.

[50] Rowley JC, Moran DT (1975). A simple procedure for mounting wrinkle-free sections on formvar-coated slot grids. Ultramicroscopy.

[51] Reynolds ES (1963). The use of lead citrate at high pH as an electron-opaque stain in electron microscopy. J Cell Biol.

[52] de Groot PW (2004). Proteomic analysis of Candida albicans cell walls reveals covalently bound carbohydrate-active enzymes and adhesins. Eukaryot Cell.

[53] Wiśniewski JR, Zougman A, Nagaraj N, Mann M (2009). Universal sample preparation method for proteome analysis. Nat Methods.

[54] Livak KJ, Schmittgen TD (2001). Analysis of relative gene expression data using real-time quantitative PCR and the 2(-Delta Delta C(T)) Method. Methods.

[55] Rønnest NP, Stocks SM, Lantz AE, Gernaey KV (2012). Comparison of laser diffraction and image analysis for measurement of Streptomyces coelicolor cell clumps and pellets. Biotechnol Lett.

