# Supplementary material for: Altered secretion patterns and cell wall organization caused by loss of PodB function in the filamentous fungus Aspergillus nidulans
Source: Sci Rep. 2018 Jul 30;8:11433. doi: 10.1038/s41598-018-29615-z (PMC6065416; doi:10.1038/s41598-018-29615-z)
Supplement: Supplementary file 1 — Supplementary information [file 41598_2018_29615_MOESM1_ESM.doc]

**Altered secretion patterns and cell wall organization caused by loss of PodB function in the filamentous fungus *Aspergillus nidulans***

Karthik R. Boppidi^1^, Liliane Fraga Costa Ribeiro^1^, Sirasa Iambamrung^1^, Sidney M. Nelson^1^, Yan Wang^2^, Michelle Momany^3^, Elizabeth A. Richardson^4^, Stephen Lincoln^5^, Ranjan Srivatsava^5^, Steven D. Harris^6^, Mark R. Marten^1, *^

1. University of Maryland - Baltimore County, Department of Chemical Biochemical and Environmental Engineering, Baltimore, MD, USA.
2. University of Maryland – College Park, Department of Cell Biology and Molecular Genetics, College Park, MD, USA.
3. University of Georgia, Fungal Biology Group and Department of Plant Biology, Athens, GA, USA.
4. University of Georgia, Georgia Electron Microscopy, Athens, GA, USA.
5. University of Connecticut, Department of Chemical and Biomolecular Engineering, Storrs, CT, USA.
6. University of Manitoba, Department of Biological Sciences, MB, Canada.

**^*^** Corresponding author. Email: [marten@umbc.edu](mailto:marten@umbc.edu) Phone: (410) 455-3439

**Supplementary information**

**Supplementary figure S1**

**
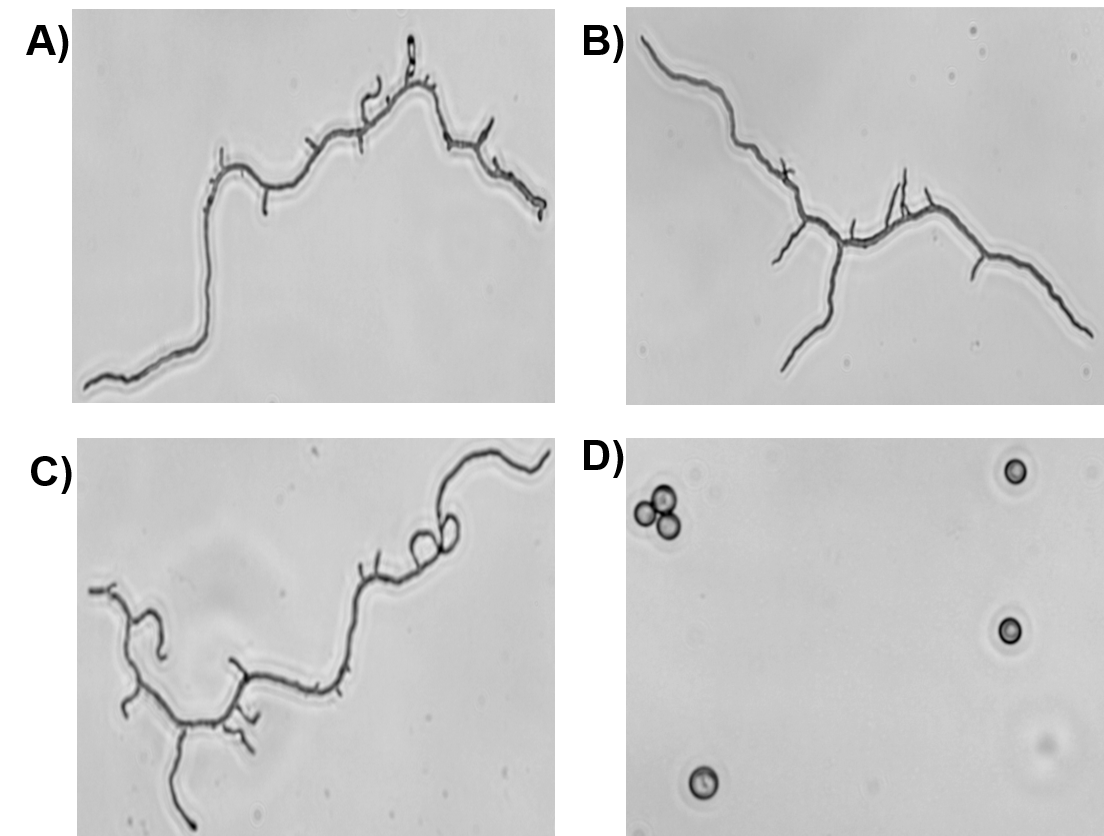
**

**Supplementary figure S1: Phenotypic impact of the temperature sensitive (Ts) *podB1* mutation.**  Microscopic images of fungal strains grown at 28^o^C and 42 ^o^C. Strain A28 (control) shows polar growth with developed germ tubes at both 28^o^C *(A)* and 42^o^C *(B).* ASH83 (podB mutant) also shows polar growth with developed germ tubes when grown at 28^o^C *(C)*. However, ASH83 shows isotropic growth, with no germ tube development, when grown at 42^o^C *(D)*.

**Supplementary figure S2**


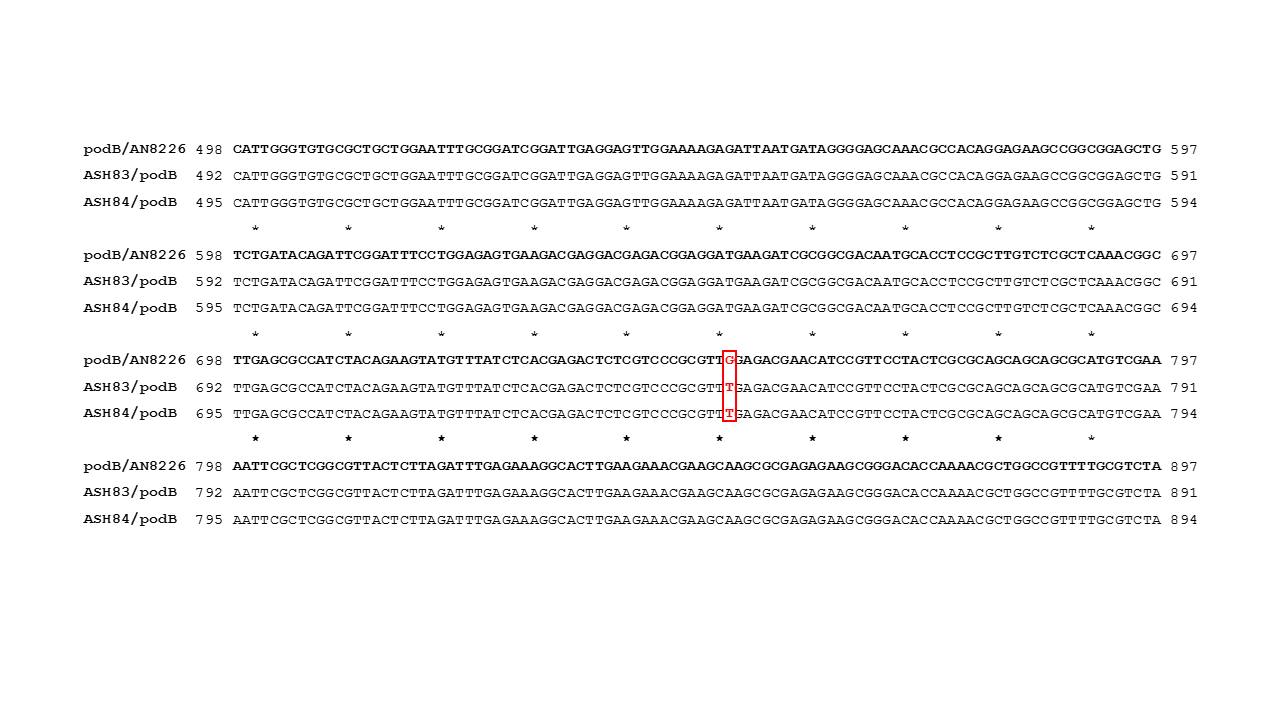


**Supplementary figure S2: Genotype of the *podB1* mutation.** Resequencing of the podB gene revealed that the G to T mutation at nucleotide #751 is the cause for the Ts phenotype of both ASH83 and not the mutation at nucleotide # 670 as previously reported^15^.

**Supplementary figure S3**

**
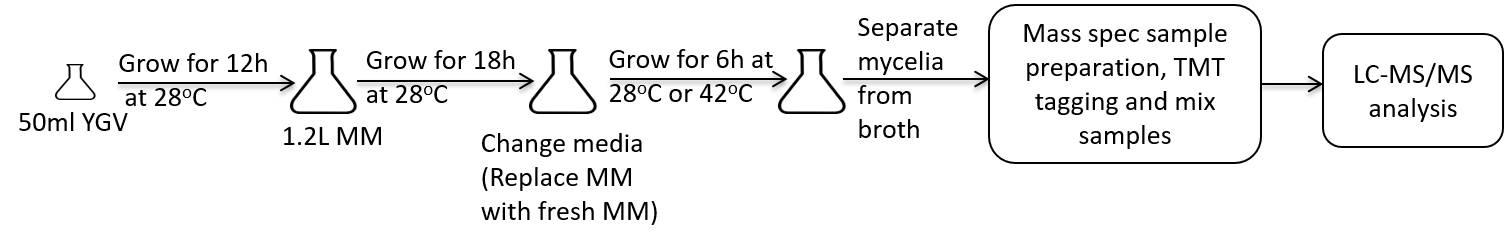
**

**Supplementary figure S3:** Strategy used for proteomics of the secretomes of both ASH83 and A28 at both PT and RT.

**Supplementary figure S4**

**
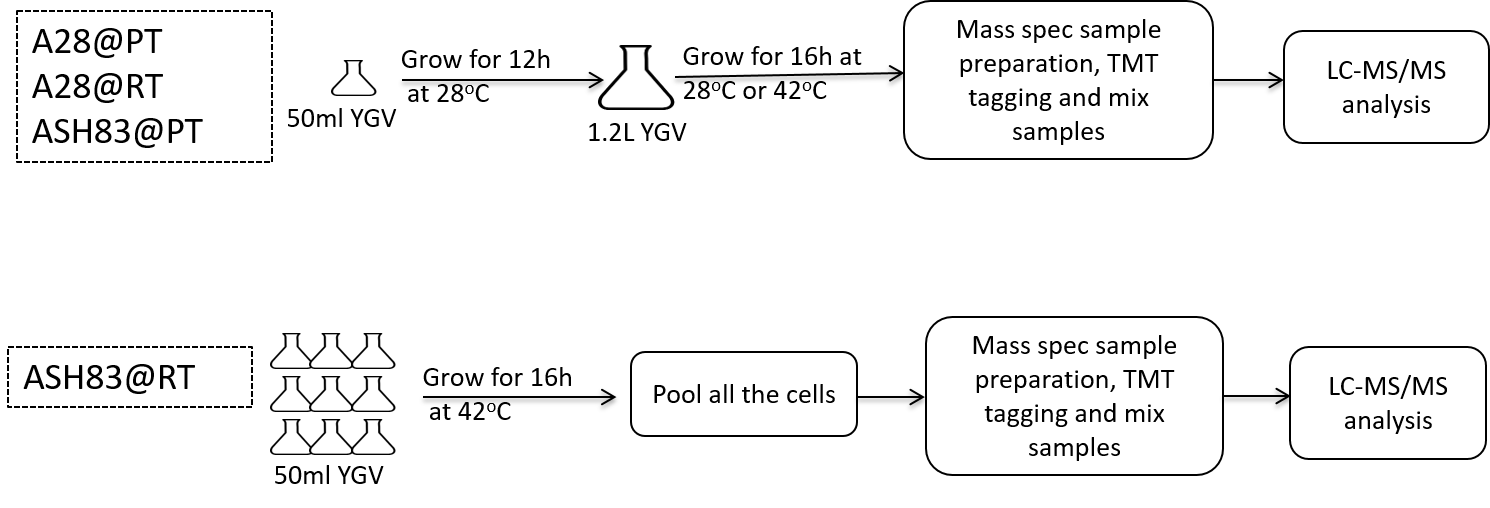
**

**Supplementary figure S4:** Strategy used for proteomics of the cell wall fraction of A28 at both PT and RT (top). Strategy used for proteomics of the cell wall fraction of A28 at both PT and RT (bottom).

**Supplementary figure S5**


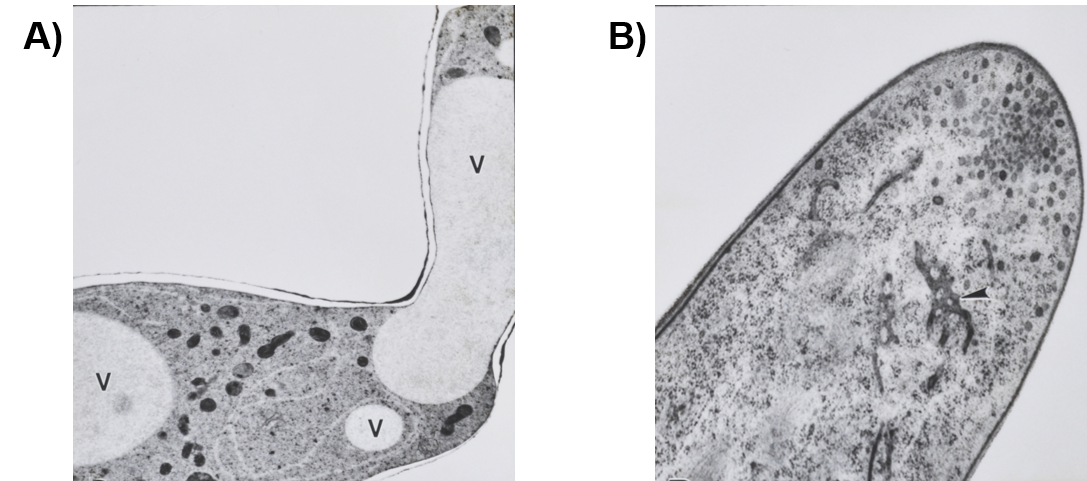


**Supplementary figure S5. Effect of Ts podB mutation:** TEM images of ASH83 incubated at 30^o^C for 11h followed by incubation at 42^o^C for 5h (A and B). The hyphal tip is shown in B. Note the normal appearance of the vesicles at the tip (Spitzenkorper). Scale bar = 1μm (A) or 0.5μm (B). V marks vacuoles; arrowhead marks golgi body.

Supplementary Table S1: Components of Vitamin mix.

| **Component** | **Amount to add to 100ml water** |
| --- | --- |
| H_2_O (d.i.) | Up to 100 mL |
| Biotin | 10 mg |
| Pyridoxin HCl | 10 mg |
| Thiamine HCl | 10 mg |
| Riboflavin | 10 mg |
| Para-aminobenzoic acid (PABA) | 10 mg |
| Nicotinic acid (or niacin) | 10 mg |

Supplementary Table S2: Components of trace elements mix.

| **Component** | **Amount to add to 100ml of water** |
| --- | --- |
| ZnSO_4_ · 7H_2_O | 2.2 g |
| H_3_BO_3_ | 1.1 g |
| MnCl_2_ · 4H_2_O | 0.5 g |
| FeSO_4_ · 7H_2_O | 0.5 g |
| CoCl_2_ · 6H_2_O | 0.16 g |
| CuSO_4_ · 5H_2_O | 0.16 g |
| (NH_4_)_6_Mo_7_O_24_ · 4H_2_O | 0.11 g |
| EDTA | 5.0 g |
| H_2_O (d.i.) | Up to 100 mL |

Supplementary Table S3: Nitrate salts used in the media.

| **Component** | **Amount per liter of media** |
| --- | --- |
| NaNO3 | 6 |
| KCl | 0.52 |
| KH_2_PO4 | 0.815 |
| K_2_HPO4 | 1.045 |
| MgSO4·7H2O | 0.52 |
| H_2_O (d.i.) | up to 1L |

**Supplementary Table S4: Primers used for amplification of genes in qPCR.**

| **Primer** | **Sequence** |
| --- | --- |
| bipA - Forward | GTGAAGTCGAGAAGGCCAAG |
| bipA - Reverse | TACGAGTGGAACCACCAACA |
| clxA - Forward | GTGAAGTCTCCGGATGTGGT |
| clxA - Reverse | GGTCCAGATCTCGAAACCAA |
| Actin - Forward | TACTCCGTCTGGATCGGTGG |
| Actin - Reverse | CTTGCGGTGGACGATCGAAG |
